# Death in worship places: Evaluating the roles of religious organisations and state governments in reducing the risks of religious disaster

**DOI:** 10.4102/jamba.v13i1.1042

**Published:** 2021-07-12

**Authors:** Helena van Coller, Idowu A. Akinloye

**Affiliations:** 1 Faculty of Law, Rhodes University, Grahamstown, South Africa; 2 Faculty of Law, Ajayi Crowther University, Oyo, Nigeria

**Keywords:** religion, religious disaster, accidents, injury, death

## Abstract

The numbers of accidents and disasters resulting in injury and death of the faithful in religious buildings in many parts of the world are on the increase in recent years. Interestingly, the citizens of the countries where most of the cases are reported are overtly religious and manifest their religiosity by attending religious activities in religious buildings. This, therefore, heightens the impact of a disaster, such as where there is a religious building collapse or a stampede. The attendant social, legal and economic effects of such disasters on religious organisations, religious faithful and society thus necessitate the study. This article critically examines the roles of religious organisations and state governments in reducing the risks of avoidable disasters in religious buildings. It evaluates the reports of two instances of church building collapses in Nigeria as case studies. This article observes that many religious organisations do not have effective risk and safety policies to reduce their exposure to religious disasters. It also observes that the state is ineffective in enforcing building standards. It argues that religious organisations and the state owe a legal duty to protect the lives and guarantee the safety of the faithful against the tragedy that may occur in worship places, and where this duty is breached, and a victim suffers harm, a right to damages will accrue. It concludes that although a religious organisation may not be able to stop all such disasters, having an effective disaster risk policy can assist in reducing the occurrence of avoidable mishaps in religious buildings.

## Introduction

A survey of the media suggests that a number of religious buildings and worship places in some parts of the world have become unsafe in recent times. Religious buildings and worship places in this regard refer to places where a religious community gathers to perform religious activities, such as Christian church buildings, mosques, temples, prayer grounds, pilgrim sites and synagogues. These places are increasingly becoming unsafe for worshippers as they suffer injuries and lose their lives resulting from a religious building collapse, stampede, amongst others. In 2005, Hammoudi drew the attention that an elevator had become a major death trap for those who visited Mecca for Islamic pilgrimage (Hammoudi 2005). Hammoudi’s observation became a global concern when in 2015, a crawler crane at the Grand Mosque in Mecca collapsed, allegedly killing 107 people and injuring several others (Karimi, Ellis & Hanna 2015). Less than 2 weeks after the incident of the crane collapse, another major religious disaster struck Mecca, resulting in the death of over a thousand pilgrims, whilst hundreds got injured during a stampede (Gambrell & Batrawy 2015). Similarly, in December 2018, about three worshippers were reported to be dead and nine injured in a stampede at the Enlightened Christian Gathering Church in South Africa (Sicetsha 2018); in October 2020, 22 worshippers were reported to be dead and several others injured in a church building collapse in Ghana (Aljazeera 2020). And very recently, a graduating student from the Department of Human Resources Management of the Uganda Christian University, Kampala, died following a church building collapse (Soyoye 2021). The reports of religious disasters in Nigeria are more precarious for the country has witnessed intermittent collapses of religious buildings, resulting in many deaths and injuries (Fowode 2016; Ogundele 2018). As will soon be discussed, no less than three religious buildings have reportedly collapsed in the country in the last half a decade. The above reports overtly reveal more reported cases of ‘church’ building collapse than mosques or any other religious worship places.

The issue of religious disasters is worrisome because the citizens of the countries where the occurrences are prevalent are widely known to be overtly religious and often manifest their religiosity by attending religious activities in religious buildings. The concern over religious disaster is heightened because a huge number of faithful pilgrims who may assemble in a worship place for a religious ritual can be susceptible to harm if a religious mishap occurs. Some questions, however, arise: what role does a religious organisation need to play in safeguarding the lives and health of the faithful pilgrims who are present in a religious building? What social and legal implications do religious disasters pose for religious organisations and society? What practical steps religious organisations should take to reduce their exposure to legal risks that may arise from religious disasters and to prevent avoidable religious disaster? What is the role of the state in reducing disasters and accidents in religious buildings? This article attempts to answer the above questions.

It is acknowledged that all the cases and countries that have experienced religious disasters may not be effectively examined in an article of this nature. Thus, two examples of recent religious disasters that involved the collapse of church buildings in Nigeria are used as case studies. In order to answer the above questions, the article uses a desktop research methodology and critical and analytical research approaches to offer an insight into the governance, legal, financial and corruption dimensions to who is responsible for disaster risk management of religious buildings, rather than the structural or faith dimension. The data for this study are primarily sourced from government white papers and corona court’s report on the collapsed church buildings in Nigeria, media reports, legislation like the Nigerian 1999 Constitution, court judgements, religious books like Bible and Qur’an and other related literature, particularly those relating to disaster risk management. From the analyses of the data, the study observes a failure of the regulatory agencies to effectively implement the building regulations and standards, particularly as it relates to seeking building approvals and permits.

The article is structured as follows: the first section examines Nigeria’s religious demography in order to contextualise Nigerians’ religiosity. This is followed by the examination of two cases of church buildings collapse in Nigeria. Thereafter, some legal and social implications that religious disasters may pose for religious organisations are examined. This is then followed by a critical review of the internal policies of the churches that experienced the building collapse, particularly as it relates to the construction and maintenance of religious buildings. It thereafter critically evaluates the roles of the state in enforcing building standards. The article concludes by arguing that religious organisations must ensure that their buildings comply with regulatory standards, and that their governance mechanisms cater for risks relating to the construction of religious buildings. It further recommends that the statutory provisions relating to state obligation in enforcing building standards are amended to make state agencies and officers liable where they are found not to have performed their statutory duties, which resulted in harm to people. It argues that although a religious organisation may not be able to stop all disasters that may occur, having an effective disaster risk policy can assist in reducing the occurrence of avoidable mishaps in religious buildings.

## Religious demography in Nigeria

Religion plays a pivotal role in Nigeria and in the daily lives of her citizens (Kitause & Achunike 2013; Vaughan 2016). There are three dominant religions in the country – Christianity, Islam and traditional religion (Nwauche 2008; Peel 2016; Stonawski et al 2016). There are also a few numbers of non-religious (eds. Grim et al. 2017, Stonawski et al. 2016) people. The United States quoting the 2012 survey by the Pew Research Center’s Forum on Religion and Public Life estimated Nigeria’s population to be 49.3% Christian and 48.8% Muslim. The remaining 2% belongs to either traditional religion or no religion (eds. Grim et al. 2017; U.S. Department of Sate 2016). This finding seems to confirm the widely held view that Nigeria and other African countries are highly religious (Agbiji & Swart 2015; Akpanika 2017; Mbiti 1969). Some scholars, such as Plaatjies-Van Huffel (2019) and Guiora (2017), and jurists like Sach (*Christian Education South Africa v Minister of Education 2000*), attribute the motivation for this religiosity to the desire for self-realisation and the search for true meaning in life. However, other scholars and commentators argue that the varying degrees of challenges that people face, such as diseases, insecurity, poverty and unemployment, are what makes many to turn to religion for succour (Fraser 2016; Miller 2014; Rees 2009). A poll by Gallup appears to confirm the latter position that religiosity is highest in the world’s poorest countries (Crabtree 2010). In the same vein, Magbadelo (2014), a Nigerian researcher of political and economic relations, posited that ‘the socio-economic and political adversities in the country provide a fertile ground for the planting, germination, growth and balkanisation of all forms of religion’, and that ‘the zeal of Nigerians for religion, like wine, waxes very strong with age’.

Akin to the above is the current ongoing debate on commercialisation of religions. It is argued that the increasing rate of proliferation of religious organisations is propelled by the economic gains that religious leaders seek to benefit (Ibrahim 2013; Olusegun 2020). Thus, the religious actors seek to build quick and huge worship places at a geometric and highly competitive rate in order to attract more faithful devotees to achieve their economic agenda.

The point is that the increasing numbers of Nigerians turning to religion, as well as the commercialisation of religion, are influencing the rise in the numbers and sizes of religious buildings and worship places (Adegoriolu, Idowu & Oyelana 2018; Akintola 2004). For instance, churches and mosques that could accommodate thousands of worshippers in a single service have geometrically increased in the last decade. For example, the Apostolic Faith Church, Lagos encompasses 100 000 worshippers (Eyoboka & Latona 2011). The Living Faith Church, Ota, also embraces 50 000 worshippers, and the church has laid the foundation of another church building that would cover 100 000 congregants (Brown 2011; Olofinjana 2011). The Redeemed Christian Church of God recently commissioned an auditorium in Ogun that encompasses over a million. The Central Mosque, Ilorin, Kwara State also cover 20 000 worshippers.

## Church building collapses

As the number of religious buildings seems to increase in Nigeria, so also the incidence of religious disasters, particularly church building collapses. Both the mainline churches (i.e. foreign originated churches such as Catholic and Anglican churches) and African Independent churches (African indigenous established churches) are experiencing these collapses. Commenting on the recurrence of church building collapses in the country, a Nigerian health and safety practitioner, Fowode (2016:1), observes:

Every year, a number of accidents occur in churches, church halls, churchyards and grounds. Not only does this cause fatality, but also pain and suffering to those people who are injured, it has often resulted in serious disruption to the smooth running of the church and its various activities. Looking at the records of fatalities that have resulted from the buildings collapse in Nigeria, it is obvious that collapse of religious building produces more casualties due to the huge number of people assembled at a particular time especially during weekly church services.

As several people are present in a religious building when holding religious services, many suffer injuries and losses in the case of a mishap. As mentioned earlier, two remarkable cases of church building collapse are evaluated. The first relates to the collapse of a building at the Synagogue, Church of All Nations (SCOAN), Lagos State, and the second is the collapse of the Reigners Bible Church, Uyo, Akwa Ibom. These two cases are of significance and have been chosen as case studies because of the huge casualty being reported, the wide publicity of the incident and the unique responses of the government after the incident.

### Synagogue, Church of All Nations

On 12 September 2014, a five-storey building on the premises of the SCOAN that is used to house mostly foreign guests collapsed. About 116 persons were confirmed dead in the disaster, 85 of whom were South African citizens, 22 were Nigerians, two were Togolese and two were Beninoise (Abdulah & Madukwe 2015). The Lagos State government in terms of the *Lagos State Coroner System Law No 7 of 2007* set up a Coroner Court (the Coroner) to investigate the cause of the building collapse. The Senior Pastor of SCOAN, Pastor TB Joshua and the Church authorities, instituted a series of suits at the High Court of Lagos State to quash the jurisdiction of the Coroner (Adebowale 2016). However, these suits were dismissed for a lack of merit. In its report, the Coroner indicted SCOAN and held, *inter alia*:

Building permits/approval was not obtained in respect of the collapsed building. The foundation failure was a remote cause of the collapsed building. The church must be investigated and prosecuted for not obtaining the relevant approval before embarking on the construction of the building … The church was culpable because of criminal negligence resulting in the death of the victims. (Abdulah & Madukwe 2015:1)

This report from Coroner further confirmed what the Lagos State Commissioner of Planning reportedly told the journalists that as there was no record of an approved building plan for the collapsed building in his office, the building was not vetted by the relevant state authority for approval (Akoni & Asomba 2014; Ukah 2016). Ukah (2016) observed further that the collapsed building was originally a two-storey building, which was later raised to six in order to accommodate the growing influx of international miracle and prophesy seekers. The SCOAN has appealed the Coroner’s judgement, and the suit is now before the Court of Appeal in Lagos.

### Reigners Bible Church

On 10 December 2016, the Reigners Bible Church building, which can hold 5000 congregants, collapsed during the consecration of the General Overseer of the Church, Apostle Dr Akan Weeks (Dr Weeks) as a bishop. At least, 50 people were reported dead in the disaster (Guardian 2016). However, some reported the numbers as high as 160 (Onyanga-Omara 2016). The Akwa Ibom state government, one of the 36 federating states in Nigeria, in terms of the Commission of Inquiry Law of 2000 of the state, set up a Commission of Inquiry (the Commission) and charged the Commission to investigate the circumstances that led to the disaster; identify any person, institution or authorities responsible either remotely or immediately for the collapse of the building; and recommend appropriate sanctions and actions against all persons found culpable in the church building collapse (Akwa Ibom 2016). In its report, the Commission observes that ‘both the design and erection processes of the building were not properly handled, which led to the structural element being overloaded, resulting in the collapse of the building’ (Akwa Ibom 2016). It further found Dr Weeks to be immediately responsible for the church building collapse. Specifically, the Commission noted that Dr Weeks:

- illegally built the church on government land without seeking and obtaining approvals
- employed and relied on quacks to construct and supervise the building project
- unduly interfered in some critical aspects during construction of the building to an extent that he ordered for the removal of the temporary support system (Central Scaffold) supporting the drum of the steel roof and deletion of the basement floor during construction against all professional advice (Akwa Ibom 2016).

Based on the above-mentioned findings, the Commission found Dr Weeks to be culpable for failing to observe the basic procedures for building and undue interference in the construction process and recommended that the Ministry of Justice take appropriate action against him (Akwa Ibom 2016).

## Legal and social implications for religious organisations

A religious building collapse may pose economic, legal and social implications for religious organisations. Besides the loss of lives and economic resources that would be suffered by persons and religious organisations in the case of a collapsed religious building, a religious organisation may arguably be further exposed to lawsuits and loss of reputation (Ede 2010). A pertinent question that may be asked is whether a religious organisation in Nigeria can be held liable for its negligence, resulting in injury and death of worshippers in a religious building? Although there is no Nigerian judicial decision known to the authors where a religious organisation was held negligently liable for harm or loss suffered by worshippers, it may be argued that a religious organisation can be held liable in delict where it is found to be negligent or in statutory breach in the construction or maintenance of a religious building, which later collapsed and caused harm to many. The decision of Supreme Court of Nigeria in *Anyah v. Imo Concorde Hotels (2002)* outlined the following ingredients for negligent liability:

The tort of negligence arises when a legal duty owed by the defendant to the plaintiff is breached. And to succeed in an action for negligence, the plaintiff must prove by the preponderance of evidence or balance of probabilities that – (a) the defendant owed him a duty of care; (b) the duty of care was breached; (c) the defendant suffered damages arising from the breach. (p. 16)

In the light of the above, an injured person would have the right to institute a civil action to claim against a religious organisation for damages where he is able to prove that the religious organisation owed him a duty of care, that the religious organisation breached this duty and that he suffered an injury resulting from the breach (Laganparsad 2016 cited in Mathebula & Smallwood 2017). This submission is the position in some other jurisdictions like in the United States of America. Patrick Sternal (2009), an American legal practitioner submits:

It is important to remember that churches can be liable for acts of negligence, as well as more serious breaches of duty. At one time there were legal protections available for charitable and religious organizations in the form of charitable immunity statutes. These have been all but abandoned for practical purposes. While some states may still have charitable immunity statutes on the books, for the most part, charitable immunity has been abolished or reduced to a minimal shield from liability for negligence against a religious or charitable organization. (p. 47)

Although not specifically a case of a collapsed building, the American case of *Dadd v Mount Hope Church and International Outreach Ministries (2009)* serves as a good reference of a religious organisation that was held liable for its negligence for failing to safeguard the life and health of a worshipper. In this case, a Michigan appellate court upheld the jury’s negligence award to a church member who sued her church and pastor for injuries and loss she suffered when, upon responding a call to the altar, ‘slain in the spirit’ and collapsed to the floor, injuring herself. Whilst the court acknowledged that the defendant church does not have a duty to provide an usher for every person who attends services, the court held that the church was aware of the possibility of injury to those who answered the ‘altar call’ and should, therefore, supposed to have ushers in place to assist participants at such ‘altar call’. The court held that the church had a duty in this situation to assist those who participate in the altar call, and thus, the church’s failure to do so in this situation was a breach of its duty of care (Sternal 2009). It may be argued, however, that a religious organisation would be absolved of liability where it is found to have taken the necessary steps and implement necessary or reasonable safety measures.

Besides the civil liability, the State may bring a criminal charge against individuals as well as the leaders of a religious organisation for a statutory breach, leading to a religious disaster (The Civil Society Network against Corruption [CSNC] 2017). For instance, as it has been observed in the above-mentioned case studies, the Coroner and the Commission recommended that the churches and their leaders be prosecuted. Where a religious organisation faces a lawsuit arising from its negligence, it may further have implications for its reputation, governance, sustainability and growth (Akinloye 2019).

## Governance issues arising

Whilst a building collapse may be caused by a natural occurrence, such as earthquake, flooding and wildfire, the findings reveal that most of the building collapses reported in Nigeria are attributable to laxity in the construction processes (Ede 2010; Fagbenle and Oluwunmi 2010; Olajumoke et al. 2009; Oloke et al. 2017). The reports of the Coroner and the Commission corroborate these findings. It may, therefore, be indicated that perhaps most of the losses resulting from the collapse of buildings in Nigeria could have been avoided, but for the laxity in the construction and maintenance of the religious buildings. From the findings of the Coroner and the Commission, some important internal policy concerns could be further identified, which implicate the two churches in the construction of their religious buildings. These concerns are discussed in the following subsection.

### Absence or inefficiency of a church building committee

All through the Commission’s report on the Reigners Church building collapse, it is observed that no mention was made regarding the role played by the project or building committee of the church during the construction activities. It appears that the church does not have one, and if it does, then, the committee is passive. It is submitted that a committee, probably a building or project committee or other similar bodies of a religious organisation, should on behalf of the church oversee the building of such a size and magnitude. Such a committee should consist of professionals whose jobs are related to building and property development, such as town planners, builders, architects, safety advisers, paramedics, engineers and lawyers (Churches by Daniels 2018; Foreman 2020). If such a committee was in existence, perhaps it would have been able to advise the church appropriately. The committee would probably have interviewed and related with those who were hired for building the church. The Commission further observed that the professionals employed in the building of the Church were incompetent. For instance, the Commission noted, ‘one Mr. Anietiem Ndarake fraudulently held himself out as an architect and assumed the responsibility of a project manager, which he lacked the requisite knowledge and experience to handle’. (Akwa Ibom 2016). Perhaps, this incompetence of Mr. Ndarake would have been discovered if the members of the committee who have insights in constructions had engaged with him. Furthermore, as it is common for religious organisations to engage their members who are professionals in building constructions, maybe in order to cut costs, the church engaged the services of its congregants who held themselves as professionals; however, these congregants are incompetent. Shaibu (2013) referred to this practice as the ‘internal identification of potential suppliers among members’, whereby a religious organisation does not go by the procurement rules of allowing potential suppliers to bid, rather they find amongst their congregation a member(s) who can supply the needs of their churches. Whilst a religious organisation may engage or hire its members to construct its building (Churches by Daniels 2018), the argument is that religious organisations must not sacrifice the needed professional competency on the altar of religious affinity and favouritism (Castrilli 2016).

### Religious leaders’ influence

Akin to the observation made above is the fact that religious leaders do unduly interfere with the implementation of governance mechanisms of the religious organisations relating to the construction of worship places, even where they do not have expertise. From the Commission’s report, Dr Weeks apparently took over what ordinarily should be the duties of a building committee because the church appears to leave all the decisions regarding the church building to him alone. Meanwhile, there was no evidence that Dr Weeks is knowledgeable about building construction. Religious leaders and ministers should not make committees of their organisations passive. Whilst building or project committee of a religious organisation may be headed by their religious ministers, their mandate should primarily be to ensure transparency and accountability, such as in the disbursement of project funds and not to take over the professional influence of the committee. Religious leaders need not spiritualise the professional advice of a building committee of their organisations.

### Noncompliance with legal standards

Another observation that could be made is that the SCOAN and Reigners Church were alleged not to have complied with the state building regulations and codes that oblige them to obtain necessary building plan approvals and permits from the government regulatory agencies. These allegations against the churches are in breach of the law, particularly sections 30, 70 and 71 of the *Nigeria Regional Planning Act 88 of 1992 and section 28(1)* of the *Lagos State Urban and Regional Planning and Development Law, 2010,* which stipulate that a developer of any building must apply for a permit and approval of building plans. Accordingly, these churches can be held liable. This issue of noncompliance by churches seems not to be limited to Nigeria. It has also been reported in South Africa. Although unlike in Nigeria, there has not been an occurrence of a church building collapse in South Africa, the investigation conducted by Mathebula and Smallwood amongst some African Independent Churches’ buildings in South Africa reveals that none of the churches investigated complied with the required legal standards of occupational health, safety, and environmental risk assessment. The scholars observe:

The organisations that were assessed, none of them complied with health and safety standards; to them, utility took priority over safety. No one took responsibility for the health and safety of their congregants… The study also has shown the fact that churches undertake to build the structures without complying with the construction regulations. What was more disconcerting; was that people who were responsible for building these structures were not qualified builders; they participate in these projects on a voluntary basis and were never trained in hazard identification and risk assessment. (Mathebula & Smallwood 2017) (p. 926)

The inference one could draw from this is that it is possible that the churches do not have legal and safety risk policies to assess whether their actions and inactions meet the required legal standards, particularly as it relates to building constructions. Usually, a legal risk policy would identify organisation’s riskiest areas and possible actions that could make them liable and implement controls to prevent the occurrences (Welch 2011). This appears to be missing in the instant cases.

### Lack of insurance policy

Equally important is the issue that flows from paragraph 6(4) of the Commission’s report, which states *inter alia*:

The commission further recommends the payment of the sum of One Million Naira [*about 3000 US Dollars*] for the dependants or administration of the estate of the deceased and a minimum of One Hundred Thousand Naira for the injured depending on the severity of the injuries sustained. (p. 11)

It is of note that the government accepted this recommendation to pay the one million naira to the estate of each of the deceased from the Reigners building collapse (Akwa Ibom 2016). A similar recommendation was made with regard to the victims of St Paul Catholic Church building collapse in Delta State, Nigeria in September 2018 (Ogundele 2018). A report from South Africa also revealed the sufferings of the dependants of those who lost their ‘breadwinners’ to the SCOAN building collapse. This report states:

Families of victims who died when Prophet TB Joshua’s Synagogue, Church of All Nations building collapsed in Lagos, Nigeria, four years ago are still searching for answers … Amid unsettled legal processes pursued by Nigerian authorities; dozens of affected families have not found closure and continue to be sidelined by authorities close to the investigation. *Some are experiencing poverty after losing their breadwinners.* **(**Sifile & Mashaba 2018:1)

It is discernible from the above reports that the churches in these cases do not have insurance cover to cater for these kinds of occurrences in order to enable them to pay compensation to the families of those who died in the collapses. Also, the contractors hired by the churches to build the buildings appear not to have liability insurance to cover the contractor’s actions that result in damage to people or property. Meanwhile, the provision of sections 27(1) & (2) and 48(1) of the *Lagos State Urban and Regional Planning Law No. 3 of 2010* stipulate that a developer of any building above two floors must insure his or her liability in respect of construction risks when making applications for building approvals and permits. It further stipulates that ‘a developer or owner of construction involving a structure of more than two floors shall at the time of submitting his application to commence building works to the Building Control Agency submit a general Contractors All Risk Insurance Policy Certificate’. Arguably, as the possession of insurance policy is one of the requirements for granting building permits, then, the fact that there was no report that the victims of SCOAN building collapse were compensated from any insurance policy, justifies the position of the Lagos State Commissioner for Physical Planning that SCOAN did not obtain a building permit regarding the SCOAN’s building that collapsed. However, whilst the governments’ decision to aid the victims of the collapse buildings is lauded for it brings succour to the victims, the argument is that churches ought to have insurance policies that would cater for such mishaps. Additionally, it is necessary that the policies of religious organisations stipulate that only contractors that have liability insurance cover would execute their construction projects. All these observations from the activities of these churches further confirm the findings that most religious organisations and their leaders in Nigeria do not take an insurance policy to cater for an occurrence of a mishap; rather, they seem to choose to live by faith (Falana 2012; Olowokudejo 2018).

Against the backdrop of the increasing rate of religious building collapse, it is expedient that religious organisations take up a property insurance policy to protect their properties and limit their risks and losses in the event of a mishap. In fact, such a policy should form the component part of their regulatory instrument. Whilst it is accepted that having an insurance policy will not stop an occurrence of a mishap, it can limit the impact of a loss or an occurrence on both the organisations and the victims. Like any other secular institutions, religious organisations are prone to various risks for which it is wise for them to have insurance coverage.

A factor that comes to mind that may perhaps make some religious organisations unwilling to take insurance policy or be concerned about religious building collapse is the philosophical idea of ‘fatalism’. According to Maercker et al. (2019), fatalism is the propensity to believe that one’s destiny is externally determined. It is an attitude or a doctrine of resignation in the face of some future events, which are thought to be inevitable. This doctrine may be termed in a local parlance phrase as ‘what will be will be’.

However, there is a general, non-data-based speculation that fatalism occurs more in African and most countries of the Global South than in the Global North (Maercker et al. 2019). Coincidentally, the incidence of religious building collapse appears to be more in the region where fatalism is said to be prevalent. Thus, it is arguable that some religious leaders and religious faithful may choose not to take responsibility for the collapse of the religious building by relating the occurrence to their idea of fatalism.

### Why are there more incidence of Church building collapse?

As already observed, there are more reported cases of Christian church building collapse than mosques within the Nigerian and African context. This observation raises a question of whether there are scriptural, denominational or belief bases for ensuring building construction standards? A study of the Bible reveals a strong prescription of ethical standards in building construction. For instance, it is supported in the Bible that a person may be held liable for failing to take adequate necessary measures when constructing a building. Deuteronomy 22:8 stipulates ‘[i]f you build a new house, you must construct a guard *rail* around your roof to avoid being culpable in the event someone should fall from it’. From the above observations, it may be deduced that making sure a church develops an effective mechanism to guarantee safety in a church building is not merely a legal obligation but also is a biblical injunction. Similarly, in 1 Kings 5 and 6, the Bible narrated the divine prescription on the standards to be observed in the building of King Solomon’s temple in Jerusalem, also known as the First Temple that was built between 951 and 957 BCE. This temple was still standing until it was destroyed in 587 BC – meaning that the building lasted for more than four centuries.

In the same vein, the Qur’an made a similar account about King Solomon’s magnificent building. Quran 38:37 provides a vivid description of the builders and divers working for Prophet Solomon (Rashid 2020). However, beside the record of King Solomon building, the Quran seems to give less expression on architectural ideals of religion buildings. Rashid (2020) stated further:

… the prophet Muhammad and the four orthodox caliphs, Abū Bakr (632–34), ‘Umar (634–44), ‘Uthman ibn Affān (644–56) and ‘Alī (656–61), were unwilling to express religious ideals through architecture. They erected modest buildings that merely met functional needs. For example, the first mosque in Islam, built after the Prophet’s migration to Medina in 622, was no more than a courtyard surrounded by mud-brick walls. (p. 5)

Given the above observations, it may be argued that the incidence of more religious building collapses in one religious faith than another is not connected to any scriptural or belief system, rather it is caused mostly by extra-religious factors.

## Assessment of the government’s response

It is necessary to also examine the roles played by the government in relation to the two building collapses. This is essential in view of the constitutional responsibility of the government to protect the lives of the citizens. Thus, section 14 (2)(b) of the Constitution of the Federal Republic of Nigeria, 1999, stipulates that ‘the security and welfare of the people shall be the primary purpose of government’. Section 33 of the Constitution also guarantees the right to life of every citizen. In the collapses, two major steps of the government in response to the collapse of the SCOAN and Reigners Church building should be commended. The first is that after the incidence of the building collapses, the two federating state governments — Lagos and Akwa Ibom, immediately responded by setting up statutory machinery to investigate the cause of the building collapse. Secondly, the Akwa Ibom state government took a step further to provide financial support to those who suffered losses in the Reigners Church building collapse. However, except for the above two laudable points, the government failed to bring the alleged suspects to book. Primarily, religious leaders are named as principally responsible for the building collapse. Furthermore, the state can be said to contribute to the buildings collapse because of the failure in effectively implementing standards and regulations for building construction. These shortcomings are examined in the following subsection.

### The unwillingness to prosecute religious offenders

An impression could be drawn from the case of Reigners Church that the government is unwilling to prosecute the religious leaders who were identified as the principal suspects responsible for the building collapse. The Commission indicted Dr Weeks and recommended that legal action be taken against him. Although the state government noted the recommendation, it refused to accept it. The only justification given by the government for its refusal was that the facts available to her indicate that Dr Weeks relied on the professionals he engaged, and that it is the obligation of the professionals to execute care and skill in the discharge of their duties or resign their appointments where they are not allowed to do so (Akwa Ibom, White paper 2016). The worry here is that the Commission was the only body that was constituted to investigate and make a report on the building collapse. The questions that now beg the answers are, which other facts did the state government rely on to reach her conclusion? Is the government expected to make a defence for Dr Weeks?

Although there is no ready answer to the questions above, there is a general notion that some religious leaders in Nigeria are very powerful that the government cannot hold them accountable for their misdeeds. In fact, some scholars argue that because religious leaders are known to have a hold on their followers, politicians patronise them to mobilise their followers as electorates in favour of the politicians (Falola 2001; Oguntola-Laguda 2015). The fact is that Nigerians place more trust in and respect the opinions of their religious leaders than their political counterparts. An empirical study using the World Values Survey (Akinloye 2018; Annual Report of the United States Commission on International Religious Freedom 2018) confirms that 97% of Nigerians feel that religion is important to them, whereas the national government only 39% (World Values Survey 2014). This report shows the wide gap of trust Nigerians have in religious institutions than in the political government. These reports also represent a typical pattern across Sub-Saharan Africa. For instance, a South African Social Attitudes Survey by the South African Human Sciences Research Council in 2008 revealed that 83% of South Africans expressed high levels of trust in religious institutions, especially relative to politicians and political parties, the latter receiving a mere 29% approval (Human Sciences Research Council 2009; Plaatjies-Van Huffel 2019). Furthermore, some political officeholders are adherents and protégé of some of these religious leaders (Akinloye 2018). For example, Chief Olusegun Obasanjo, the former President of Nigeria, and Yemi Osinbajo, the current Vice-President of the country, declared publicly that they consulted and sought the approval of a renowned Nigerian Christian cleric, Pastor Enoch Adeboye, before assenting to take up political offices (Eyoboka & Olatunji 2017). It suffices to also note that the incumbent governor of Akwa Ibom state, Udom Emmanuel, was reportedly present with his political associates in the Reigners Church to witness Dr Weeks’s consecration when the Reigners Church building collapsed (Ekpimah 2016). On this issue of religious leaders’ influence, Adigwe (2004) stated further:

Our politicians have found it very useful, even more in recent times, to cling to one religious group or the other as if that group were the driving force, or source of their political power, while at the same time wanting all of us to believe that they strive to govern us, or are governing us in the name of God. Political aspirants … now have ‘prophets’, ‘imams’ and ‘native doctors and seers’ whom they consult regularly, especially in times of crises. (p. 252)

From the above observations, it may be inferred that the government chose not to take legal action against the revered religious leaders in order to not to embarrass or ridicule them, and also to continue to get the support of the religious leaders. Whilst this article does not criticise the degree of relationship between politicians and religious leaders; after all, sections 38 and 40 of the Nigerian Constitution grant to everyone the right to freedom of religion and association, respectively. The point is that the state has a constitutional responsibility to protect the lives and safety of the citizens and it must be seen to be committed to the fulfilment of this obligation. Therefore, the government should not shield persons who are alleged to commit crime at the expense of the other citizens.

### Ineffectiveness of the regulatory agencies

All the federating states in Nigeria have standards and regulations for building administration, construction and control. The provisions of these states’ regulations are substantially similar. Thus, for the purpose of this study, the provision of the *Urban and Regional Planning and Development Law of Lagos State 3 of 2010 (the Law)* is used as a reference. Section 1(1) of the Law establishes three agencies in the state, namely, the Lagos State Physical Planning Permit Authority, the Lagos State Building Control Agency and the Lagos State Urban Renewal Agency (the three are referred to as the agencies). In terms of section 1(2) and (3), the agencies are under the control of the Ministry of Physical Planning and Urban Development of Lagos State (the Ministry). The Ministry and the agencies are by virtue of section 2(b) of the Law charged with the responsibility of implementing policies regarding general building administration and control in the state. Section 2(k) further imposes the responsibility on the Ministry to develop a database for building construction and control in the state. Section 26 empowers the Physical Planning Permit Authority to process and issue building permits to individuals and entities, whilst section 47 empowers the Building Control Agency to enforce building control regulations, regulate and inspect building works, certify various stages of building construction and maintain such records; remove illegal and non-conforming buildings, identify and remove distressed buildings to prevent collapse; and administer building construction control in all its ramifications and cooperate with the Planning Permit Authority to achieve zero tolerance of illegal developments. Section 3(b) vested in the Ministry, the power to direct any of the agencies to seal up any premises for any alleged contravention of any physical planning or building control law and regulations for the purpose of enforcement and compliance. Section 60 also stipulates that the relevant agency may serve and implement enforcement notices (such as contravention, stop work order, quit, seal-up and demolition notices) on the owner of a building wherever any development is commenced without planning permit. The following question is relevant at this moment: given the facts of the case examples, can it be said that the Ministry and the agencies are effective in implementing the laws regarding building control?

In order to answer the above question, the statement allegedly credited to the Lagos State Commissioner of Planning that there was no record of SCOAN building approval and because of this, the building was not vetted by the relevant state authority for approval (Akoni & Asomba 2014; Ukah 2016) needs to be critically evaluated. To say the least, this statement is ridiculous and not justifiable. The question is, are the Ministry and the agencies not responsible for identifying buildings that have no plans and approval, and sanction any person or body liable for the statutory breach? The Commission and the Coroner observed that neither SCOAN nor Reigners Church sought the necessary permit and approvals regarding their collapsed buildings. It should be noted that the size and magnitude of SCOAN and Reigners Church buildings are the types that the regulatory agencies cannot claim ignorance of their constructions until the buildings collapsed. The only possible inference that could be drawn from the commissioner’s comment is the show of the ineffectiveness of the regulatory agencies and the supervisory Ministry. It is imperative that the regulatory bodies become up and doing to ensure that public buildings such as religious buildings that house a huge number of persons comply with building quality, standards and regulations.

It is apposite to note that all through the Law, there is no provision where the agencies or the Ministry is to be held liable for failing to perform their duty. This, perhaps, might be the reason for their ineffectiveness. Where a religious organisation constructs a building without the necessary approvals and the state agencies also failed to identify such illegality and take necessary legal actions, both the state and the religious organisation should be held liable if there is any mishap in such a building. This is because a person who enters a religious building would assume that the religious building has been constructed having complied with the necessary building standards and regulations, and that the state has given necessary permits and approvals before the building is put to use. Given the above observations, it is logical to conclude that the state agencies also contribute to the building collapses because of their ineffectiveness. To this end, it is expedient that the provisions of the Law are amended to impose liability on the state agencies and state officers who fail to carry out their responsibilities under the law regarding building controls.

## Summary and conclusion

This research article sets out to examine the roles of religious organisations and state government in ensuring that measures are put in place to reduce the risks of accidents and disasters that may occur in religious buildings. It is observed that there is an increase of religious disasters resulting in injuries and loss of lives, particularly in highly religious countries, such as Saudi Arabia, Nigeria and South Africa. Using two examples of a religious building collapse in Nigeria as case studies, it was discovered that although disaster may be a natural occurrence that is beyond the control of human beings, the cases examined are attributable to human faults, which make the occurrences avoidable. It is observed that most religious organisations, particularly in Nigeria and South Africa, lack the basic policies relating to disaster risk prevention and management. They rather prefer to live by faith than take necessary safety measures against the mishap. Religious organisations need to learn from the past incidents and develop policies to prevent their buildings from the risk of disasters. It may be necessary for a religious organisation to engage professionals in this regard. This is more expedient because of the lawsuits that religious organisations may be exposed to for failing to safeguard the lives of the faithful who worship in their buildings. Furthermore, the health and safety regulations apply to all worship centres, and where a church property causes harm to an individual, the church and church officials may be liable (Akinloye & Akinloye 2019). For instance, in the case of *Olatunbosun v The State (2011)*, the Nigerian Supreme Court emphasised the need for ensuring the security of persons in premises used for religious activities. The argument is not only reasonable in the context of the increased church building collapse but it is supported in the Bible that a person may be held liable for failing to take adequate, necessary steps when constructing a building. Deuteronomy 22:8 stipulates ‘If you build a new house, you must construct a guard rail around your roof to avoid being culpable in the event someone should fall from it’. From the above observation, it may be inferred that making sure a church develops an effective mechanism to guarantee safety in a church building is not merely a legal obligation but also a biblical injunction.

On the part of the state government, it was observed that state agencies have not been effective in the implementation of statutory provisions and standards relating to building constructions. Accordingly, these agencies need to become more proactive in identifying buildings, particularly those that occupy several people and ensure they comply with the regulatory standards. The state has a constitutional responsibility to safeguard the lives and health of all citizens, including those in religious buildings. Furthermore, where individuals, particularly religious leaders and state officials are alleged to have contravened the building regulations, the state should not be reluctant to prosecute them in order to serve as a deterrence to others.
